# Alone Together Then and Now

**DOI:** 10.3201/eid1510.000000

**Published:** 2009-10

**Authors:** Polyxeni Potter

**Affiliations:** Centers for Disease Control and Prevention, Atlanta, Georgia, USA

**Keywords:** Art science connection, emerging infectious diseases, art and medicine, Edgar Degas, Absinthe, nontuberculous mycobacteria, emotional isolation, Marcellin Desboutin, Ellen Andrée, about the cover

**Figure:**
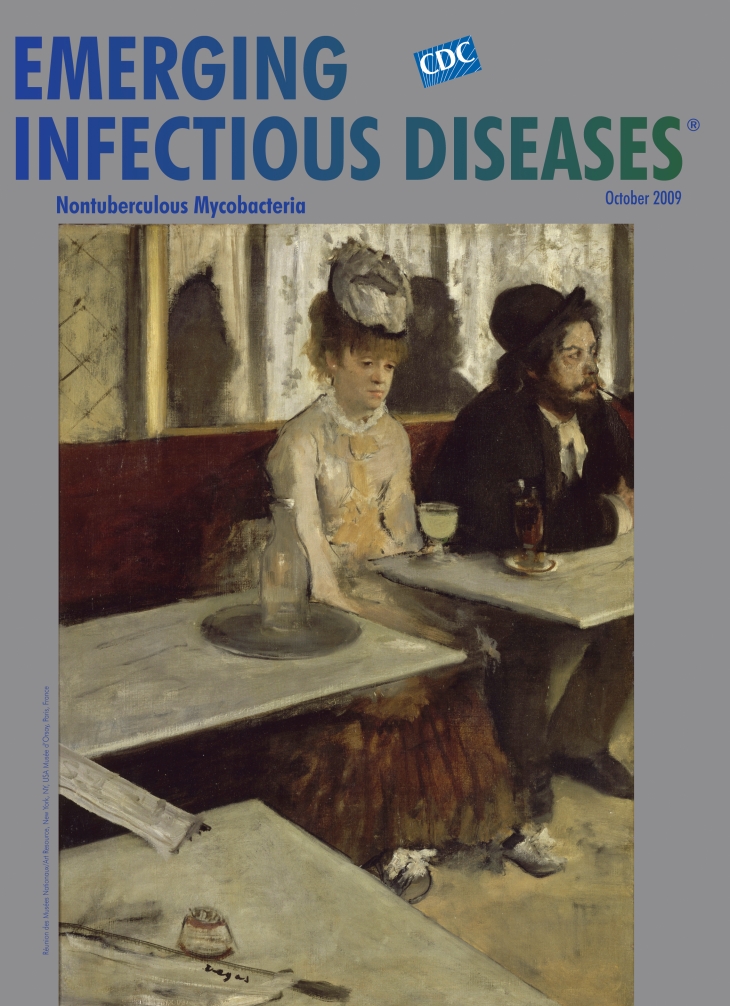
**Edgar Degas (1834–1917) Absinthe (c. 1876)** Oil on canvas (92 cm × 68 cm) Photo: Hervé Lewandowski. Réunion des Musées Nationaux/Art Resource, New York, NY, USA Musée d’Orsay, Paris, France

“Sickly, neurotic, and so myopic that he is afraid of losing his sight; but for this very reason an eminently receptive creature and sensitive to the character of things,” wrote French writer and art critic Edmond de Goncourt about Edgar Degas. The artist knew his own difficult nature. “[I have] one terrible, irreconcilable enemy,” he once admitted to Pierre-Auguste Renoir, “myself, of course.” From those who associated with him, Degas exacted an emotional toll. “There will be a dish cooked without butter for me. No flowers on the table, very little light …. You’ll shut up the cat, I know, and no one will bring a dog. And if there are women there, ask them not to put smells on themselves …. Scent, when there are things that smell so good! Such as toast, for example. And we shall sit down to table at exactly half-past seven.”

“All his friends had to leave him,” Renoir reported, “I was one of the last to go, but even I couldn’t stay till the end.” Considered a misogynist by some, Degas counted among his friends Mary Cassatt, Berthe Morisot, and leading opera divas and ballerinas of his day. Accused of being a recluse, he denied it. “I am not a misanthrope, far from it, but it is sad to live surrounded by scoundrels.” Despite his uncompromising persona, he was respected by his peers, who were afraid of him, and was popular with art critics and buyers. “I was, or appeared to be, hard with everyone, owing to a sort of tendency towards roughness that originated in my doubts and my bad temper.”

Born in Paris during the same decade as Édouard Manet, Paul Cézanne, and Claude Monet, Degas had many opportunities. His early years were privileged, though tinged by the melancholy that followed him all his life. “I was sulky with the whole world and with myself.” Under pressure he agreed to study law but soon abandoned the effort and pursued art with a fervor that convinced his father to support him, first at the Atelier Lamothe and École des Beaux-Arts and then independently in Italy, “the most extraordinary period of my life.”

Degas’ early works were historical paintings in the classical tradition. Early in his training, he absorbed the methods of Auguste-Dominique Ingres, Eugène Delacroix, and Gustave Courbet and aspired to paint like Michelangelo and Raphael. But by the 1860s, he abandoned history for scenes of everyday life. While copying a Velásquez at the Louvre, he met Manet, who became his friend and brought him into the circle of impressionist painters. Though Degas exhibited with them often, he never identified himself with the movement. “What I do is the result of reflection and study of the great masters. Of inspiration, spontaneity, temperament I know nothing.” He was not interested in the transient effects of light on landscape. He preferred painting people and abhorred painting *en plein air*. “The gendarmes should shoot down all those easels cluttering up the countryside.”

“Draw lines, young man, a great many lines,” Ingress once advised Degas, who took the comment to heart. “I always tried to urge my colleagues along the path of draftsmanship, which I consider a more fruitful field than that of color.” Always seeking perfection, he reworked every picture, even after it was sold, studying and repeating details until he had mastered and memorized them. Owners were known to chain his works to the wall. He experimented with many media, among them pastels, which he softened over steam into a paste and used over gouache and monotype prints. He disliked the shine of oil paints, so he removed the oil and applied with turpentine, often on paper rather than canvas.

Absinthe, on this month’s cover, appears to be a genre scene. But it is a portrait of Degas’ friend Marcellin Desboutin, writer, artist, printmaker, and a regular at the Café de la Nouvelle-Athènes, a meeting place for the impressionists and others in the avant garde. “I did not go to either Oxford or Cambridge,” Irish art critic George Moore said about his education, “but I went to the Nouvelle-Athènes.” Against all convention, the focal point of the portrait is a woman seated at Desboutin’s side. She is Ellen Andrée, a model who posed often for Degas and Renoir and aspired to be a serious actress, “like Sarah Bernhardt … in Phèdre.”

This painting of an unloving couple was called “the perfection of ugliness” by one critic and caused a stir when exhibited in London. “It is not a painting at all,” other critics said, “It is a novelette―a treatise against drink.” Desboutin, his elbow a wall between him and his companion, is detached, lost in thought. He is not even entirely in the picture―pipe, arm, and one leg cropped, eyes glaring off somewhere. She is precariously in center stage, her social status exposed. Pushed off one table, not quite at the next one, she sits in-between, as awkwardly positioned as her carafe.

The painting’s architecture drives the story, framing it in fresh and innovative ways. The marble tables, zigzagging across the picture, create perspective by drawing the eyes to the figures barricaded behind them, whose reflections in the mirror suggest the presence of other patrons without actually showing any. Newspapers form a bridge between the tables. The artist’s signature seems a seal of approval.

This nearly monochromatic snapshot lays bare human isolation in the midst of gaiety. Desputin’s drooping companion, propped up behind a glass of absinthe, represents women, many of them in Degas’ very neighborhood, caught in ill-fitting bohemianism, absinthe not withstanding, for as Oscar Wilde put it, “After the first glass, you see things as you wish they were. After the second, you see things as they are not. Finally, you see things as they really are, and that is the most horrible thing in the world.”

Degas’ women were often at a disadvantage, whether they sought solace in cafés, danced *en pointe*, or washed and ironed clothes in substandard conditions for meager pay. They got sick and died young. But through their drab lives, fluffy costumes, or trappings of gaiety in busy venues, the artist’s penetrating eye captured for all to see not emotional isolation alone, which had marred his own life, but its many causes: poverty, social stigma, and underlying illnesses, not the least of them tuberculosis, rampant in his day.

The costumes have changed and absinthe is no longer the drug of choice, but emotional isolation lives on as do its many causes. Therapies have curtailed tuberculosis in some parts of the world, despite the emergence of multidrug resistance, but pulmonary infections caused by nontuberculous mycobacteria are on the rise, prompting investigations and gathering of data to explore and identify what Ellen Andrée was not able to find in the bottom of the absinthe glass.
